# Le déficit immunitaire commun variable compliqué d’une amylose: à propos d’un cas

**DOI:** 10.11604/pamj.2022.42.286.29909

**Published:** 2022-08-16

**Authors:** Ibtissam Jbara, Sophia Bouamama, Fouad Haddad, Fatima Zahra El Rhaoussi, Mohamed Tahiri, Wafaa Hliwa, Ahmed Bellabah, Wafaa Badre

**Affiliations:** 1 Service d'Hépato-Gastro-Entérologie, Hôpital Ibn Rochd, Casablanca, Maroc

**Keywords:** Déficit immunitaire commun variable, amylose, dilatation des bronches, granulome, cas clinique, Common variable immune deficiency, amyloidosis, bronchiectasis, granuloma, case report

## Abstract

Le déficit immunitaire commun variable (DICV) est le plus fréquent des déficits immunitaires symptomatiques de l'adulte mais reste toutefois rare. Il est caractérisé par son spectre phénotypique extrêmement hétérogène. Nous rapportons le cas d'un patient âgé de 39 ans, qui a consulté pour des diarrhées chroniques avec une fistule anale. Le bilan biologique a révélé un syndrome inflammatoire, un syndrome de malabsorption, hypo-gammaglobulinémie à l'électrophorèse de protéines, hypo-gammaglobulinémie globale au dosage pondéral des immunoglobulines et un taux bas de lymphocytes à l'analyse des sous-populations lymphocytaires permettant ainsi de confirmer le diagnostic du déficit immunitaire commun variable (DICV) compliqué d'une amylose systémique type AA identifié par des dépôts amyloïdes sur des biopsies. L'intérêt de ce cas clinique est de ne pas méconnaitre les différentes présentations digestives du déficit immunitaire qui sont fréquentes et de l'évoquer devant une symptomatologie résistant au traitement habituel.

## Abstract (en)

Common variable immune deficiency (CVID) is the most common symptomatic immunodeficiency in adults, but it remains rare. It is characterized by its extremely heterogeneous phenotypic spectrum. We here report the case of a 39-year-old patient presenting with chronic diarrhoea with anal fistula. Laboratory tests showed inflammatory syndrome and malabsorption syndrome, hypogammaglobulinemia on serum protein electrophoresis, global hypogammaglobulinemia in weight-based assignments for immunoglobulin and a low level of lymphocytes in the analysis of lymphocyte subpopulations, thus confirming the diagnosis of common variable immune deficiency (CVID) complicated by systemic AA amyloidosis identified by amyloid deposits in the biopsies. This study highlights the importance of paying attention to common gastrointestinal symptoms of immune deficiency and to suspect it in patients with treatment-resistant symptoms.

## Introduction

Le DICV est caractérisé par un défaut de production d'anticorps responsable d'une hypo-gammaglobulinémie d'expression variable. Il est défini par une diminution d'au moins deux écarts-types des taux sériques d'au moins deux classes d'Ig, en absence d'autres causes secondaires d'hypo-gammaglobulinémie [1]. C'est une pathologie rare de révélation tardive touchant entre 1/50 000 à 1/100 000 à l'échelon mondial [1]. Les manifestations gastro-intestinales sont diverses dont l'incidence varie de 20 à 60%, parfois révélatrices de la pathologie avec certaines particularités histologiques et thérapeutiques [1]. L'évolution du DICV comme toute maladie inflammatoire chronique peut être marquée par l'apparition de complications telle une amylose qui reste rare. Nous rapportons le cas d'un homme de 39 ans qui a consulté pour une diarrhée chronique avec une fistule anale, chez qui les examens biologiques et histologiques ont confirmé le diagnostic de déficit immunitaire commun variable compliqué d'une amylose systémique type AA.

## Patient et observation

**Informations relatives aux patients (présentation du patient):** patient, âgé de 39 ans, issu d'un mariage consanguin, et dont les enfants, des jumeaux sont morts en période néonatale. Il rapporte des facteurs de risque de contage viral (rapports sexuels non protégés) et une notion de consommation de cannabis sevré il y a 2 ans. Il a été traité pour une tuberculose intestinale en 2014 retenue sur des diarrhées chroniques sans arguments microbiologiques, endoscopiques ni histologiques, traité en 2019 pour une tuberculose pulmonaire retenue sur une toux productive avec hémoptysie encore une fois sans autres arguments. Dans la même année, le patient a été opéré pour fistule anale en deux temps opératoires par fistulectomie. L'analyse histologique de la collerette chirurgicale retrouve une réaction inflammatoire granulomateuse sans nécrose caséeuse. Sur ces résultats, le traitement anti-bacillaire a été repris pour uniquement 3 mois ensuite interrompu devant la persistance des diarrhées et la réapparition d'un orifice fistuleux anal actif. Devant la suspicion de maladie de Crohn, un traitement par corticothérapie et Azathioprine a été démarré pendant 1 mois arrêté par le malade qui a été ensuite référé par le médecin traitant pour complément de prise en charge.

**Figure 1:**
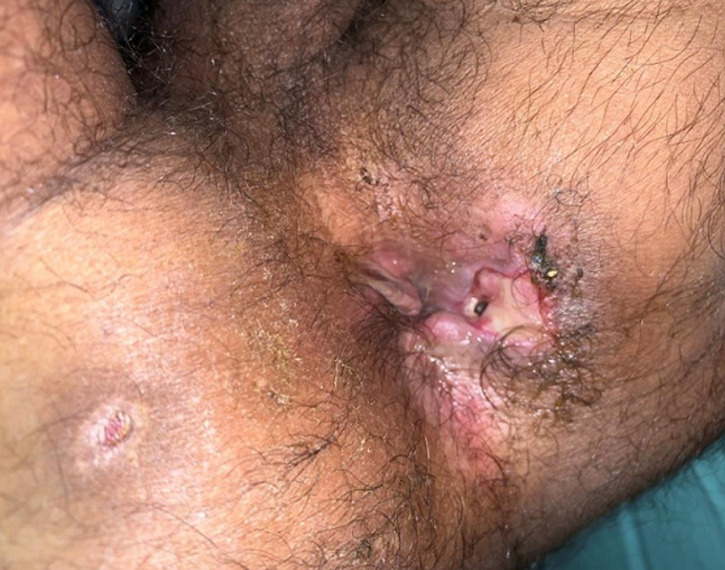
orifice fistuleux actif sur la cicatrice de l'ancienne chirurgie proctologique

**Résultats cliniques:** l'examen clinique retrouvait un patient cachectique, avec une performance statuts à 4 et un orifice fistuleux actif sur la cicatrice de l'ancienne chirurgie proctologique (Figure 1). L'auscultation pleuro-pulmonaire notait des râles ronflants diffus.

**Figure 2:**
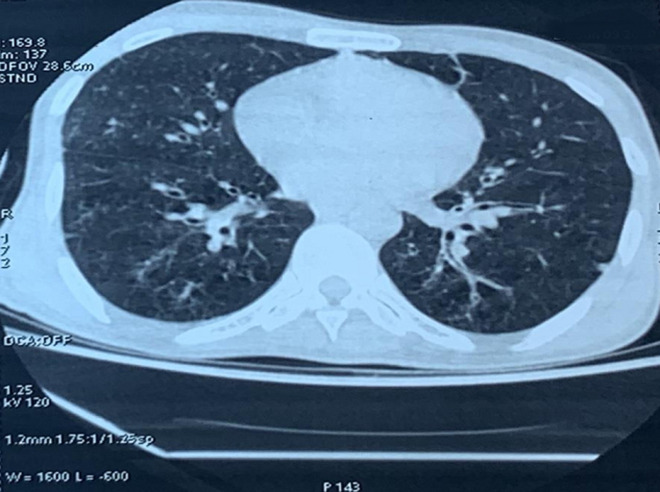
coupe scanographique montrant la fenêtre parenchymateuse du scanner thoracique montrant une dilatation des bronches bilatérales

**Démarche diagnostique:** le bilan biologique avait objectivé une anémie normochrome normocytaire arégénérative à 7,5g/dl, une leucopénie (polynucléaires neutrophiles (PNN) à 1400/mm^3^ et lymphocytes à 1630/mm^3^) et une thrombopénie à 107000. Un syndrome inflammatoire avec une protéine C-réactive (CRP) à 75mg/l et un syndrome de malabsorption (hypo-albuminémie à 21g/l et un taux de prothrombine à 66%). La coproculture et les examens parasitologues des selles étaient stériles. Le scanner thoraco-abdominal objectivait un aspect réticulo-micronodulaire diffus avec des foyers de dilatation de bronches basithoraciques (Figure 2) et montrait une hépato-splénomégalie.

A la fin de cette synthèse clinique et paraclinique, les diagnostics évoqués étaient: une tuberculose multifocale à rechute, pulmonaire, digestive et hématopoïétique écarté devant les 3 bacilles de Koch (BK) expectorations, l'intradermoréaction à la tuberculine, l'analyse du GeneXpert sur les crachats aussi bien que l'indentification du bacille de Koch par méthode de réaction en chaîne par polymérase (PCR) sur les biopsies de la reprise chirurgicale de la fistule anale et des biopsies duodénales qui étaient négatifs. Une maladie de Crohn iléo-colique avec manifestations ano-périnéales. Cependant l'iléo-coloscopie était sans particularités et la fibroscopie oeso-gastroduodénale avait montré un aspect de duodénite nodulaire avec à l'étude anatomopathologique: une atrophie villositaire sans signes histologiques évocateurs de maladie inflammatoire chronique de l'intestin. L'entéro- imagerie par résonance magnétique (IRM n'a pas retrouvé d'épaississement digestif, mais a montré un épanchement péritonéal de faible abondance non ponctionable en rapport probablement avec une entéropathie exsudative. Uniquement l'IRM pelvienne a objectivé une fistule trans-sphinctérienne type 2 de Parks compliquée de collection, drainée au bloc de proctologie par des anses élastiques.

L'étude anatomo-pathologique de la collerette retrouve des dépôts amyloïdes. La chronicité des manifestations digestives et respiratoires a fait suspecter puis confirmer: un déficit immunitaire commun variable (DICV) retenu sur une sérologie VIH négative, hypo-gammaglobulinémie à l'électrophorèse de protéines à 0,9g/l, hypo-gammaglobulinémie globale au dosage pondéral des immunoglobulines (IgA inférieures à 0,20g/l, IgM inférieures à 0,24g/l et IgG inférieures à 1,71g/l). L'analyse des sous-populations lymphocytaires a mis en évidence un taux bas de lymphocytes B (CD19) à 13c/mm^3^, lymphocytes NK à 33c/mm^3^, lymphocytes T (CD4) à 372c/mm^3^ et lymphocytes T (CD8) à 920c/mm^3^. Une amylose systémique type AA retenue sur l'identification des dépôts amyloïdes sur les biopsies des glandes salivaires, les biopsies duodénales, les biopsies coliques et intestinales, ainsi que les biopsies de la collerette chirurgicale de la fistule anale. La biopsie ostéo-médullaire était sans particularités éliminant une amylose hématopoïétique et une hémopathie maligne compliquant le tableau. Le diagnostic final retenu est un déficit immunitaire combiné variable à expression maladies inflammatoires chroniques de l'intestin (MICI) like, compliqué de dilatation de bronches et d'amylose systémique type AA.

**Intervention thérapeutique:** le patient avait bénéficié de cures d'immunoglobulines et un régime sans gluten.

**Suivi et résultats des interventions thérapeutiques:** l'évolution clinique était favorable notamment par la disparition des diarrhées et un gain de poids.

**Consentement éclairé:** le patient a donné son consentement pour la publication des informations médicales et ses photos. Il a été informé de la pertinence de son cas et l´intérêt d´en faire une publication scientifique.

## Discussion

Le déficit immunitaire commun variable (DICV) se définit par un taux d'IgG sérique inférieur à 5 g/dL, associé à un déficit en IgA et/ou en IgM, et le plus souvent une absence de réponse aux antigènes vaccinaux [2]. Il s'observe de façon égale dans les deux sexes [2]. Son expression clinique est hétérogène dominée par des infections répétées et/ou sévères le plus souvent des voies aériennes ou digestives, associée à des maladies auto-immunes ou un syndrome lymphoprolifératif [2]. Les manifestations gastro-intestinales sont très variables, fréquentes (20-60%), et peuvent être révélatrices du DICV [1]. Elles sont dominées par les diarrhées chroniques, à étiologies multifactorielles, liées à des infections chroniques surtout à Giardia lamblia mais peuvent également être dues à des atteintes spécifiques de la muqueuse intestinale comme les atrophies villositaires rapportées dans 31% des biopsies duodénales [3] pouvant simuler une maladie cœliaque [1]. La présentation digestive associée à une symptomatologie respiratoire, fait évoquer une tuberculose digestive, notamment dans notre contexte d'endémicité de cette infection bactérienne dans notre pays. Le scanner thoracique chez notre malade n'a pas montré de cavernes, cependant des foyers de dilatations de bronche. La dilatation des bronches est une expression radiologique habituelle, retrouvée chez 18 à 68% des patients atteints de DICV [4]. Cette DDB est le plus souvent bilatérale et diffuse, plutôt kystique, sans prédominance apicale ou basale [4]. Sa fréquence est accrue lorsqu'il existe un déficit complet en IgA, un taux bas d'IgM ou un déficit profond en lymphocytes B mémoires [4], le cas de notre malade. Ces foyers de dilatations des bronches (DDB) peuvent se surinfecter, mais aussi être le siège de l'érosion des vaisseaux satellites aux ramifications bronchiques responsable de la survenue d'hémoptysies [4]. Rappelons que notre malade rapporte des hémoptysies dans ses antécédents.

Certains tableaux peuvent également se rapprocher de véritables maladies inflammatoires chroniques de l'intestin. L'analyse histologique de la muqueuse colique, iléale et/ou gastrique peut retrouver un infiltrat à cellules mononuclées du chorion, l'augmentation du nombre de macrophages, la distorsion des cryptes et un infiltrat à polynucléaires neutrophiles avec lésions de cryptites et d'abcès cryptiques [5]. Néanmoins l'absence de plasmocytes dans le chorion, de granulome et de toute autre cellule géante permettent de distinguer le DICV des MICI. De plus, le profil des cytokines sécrétées dans le chorion semble différent [5].

Le diagnostic d'un DICV comporte une électrophorèse des protides, réalisée en premier objectivant une hypo-gammaglobulinémie, un dosage pondéral des Ig, une numération des lymphocytes B circulants et un phénotypage lymphocytaire (Lym T (CD3, CD4, CD8) et Lym B (CD19, CD20, CD27)) permettent ensuite d'identifier le sous-type [1]. L'évolution du DICV comme toute autre pathologie inflammatoire ou infectieuse peut être compliquée d'une amylose secondaire type AA. C'est une maladie systémique liée au dépôt de la protéine sérique de l'amyloïde A (SAA), insoluble, dans les différents tissus de l'organisme aboutissant à l'altération de leur fonctionnement. Ce dysmétabolisme est secondaire à une inflammation chronique dont les étiologies sont multiples (maladies inflammatoires rhumatismales et digestives chroniques, les maladies auto-inflammatoires, les la tuberculose [6]). Quels que soient les organes atteints le diagnostic est histologique mettant en évidence des dépôts d'amyloïdes par coloration rouge Congo avec biréfringence jaune-verte en lumière polarisée [7]. Parfois les dépôts amyloïdes sont identifiés de façon fortuite dans des prélèvements biopsiques digestifs (muqueuse gastrique, duodénale, colique) [8]. Tel était le cas chez notre patient, où l'analyse histologique des biopsies coliques étagées et duodénale a objectivé des dépôts amyloïdes type AA, recherchés systématiquement devant la chronicité du tableau clinique.

Le traitement du DICV est un traitement substitutif à vie en immunoglobulines [1, 2]. Il repose sur des perfusions d'Ig intraveineuse et plus récemment sur des perfusions à administration sous-cutanée [1]. Il n'existe pas de protocole consensuel, mais il est recommandé de commencer par un bolus d'Ig intraveineuse à la dose de 1 mg/kg, suivi soit par des perfusions intraveineuses mensuelles à la dose de 0,4 g/kg, soit par des perfusions sous-cutanées hebdomadaires à la dose de 0,1 à 0,2 g/kg [1]. L'objectif est d'atteindre un taux résiduel de gammaglobulines sériques de 5 g/l [1]. Le traitement des manifestations digestives est spécifique à chaque situation. Les épisodes infectieux bactériens doivent être traités de façon classique par une antibiothérapie [2]. En présence d'atrophie villositaire, un régime sans gluten pourrait être indiqué. En effet, des rares observations d'association du DICV à une maladie cœliaque, pour laquelle le régime sans gluten à un effet favorable ont été rapportées [1]. Comme c'est le cas de notre malade la symptomatologie digestive s'est nettement amélioré après le régime sans gluten avec disparition des diarrhées et un gain de poids.

## Conclusion

Une méconnaissance des praticiens de ce mimétisme phénotypique des maladies dysimmunitaires, masqué par les tableaux infectieux et les pathologies inflammatoires, amène à des diagnostics tardifs avec des complications potentiellement létales, à savoir l'amylose systémique, il faut savoir l'évoquer devant une symptomatologie résistant au traitement habituel bien conduit.
